# Pleiotrophin Expression and Actions in Pancreatic β-Cells

**DOI:** 10.3389/fendo.2022.777868

**Published:** 2022-02-18

**Authors:** Julio Sevillano, Aileen Liang, Brenda Strutt, Thomas G. Hill, Sandra Szlapinski, Maria Pilar Ramos-Álvarez, David J. Hill

**Affiliations:** ^1^ Department of Chemistry and Biochemistry, Facultad de Farmacia, Universidad CEU San Pablo, CEU Universities, Madrid, Spain; ^2^ Lawson Health Research Institute, St Joseph Health Care, London, ON, Canada; ^3^ Department of Physiology and Pharmacology, Western University, London, ON, Canada; ^4^ Department of Medicine, Western University, London, ON, Canada

**Keywords:** pleiotrophin, β-cell, islet of Langerhans, pancreas, RPTP β/ζ, DNA synthesis

## Abstract

Pleiotrophin (PTN) is a heparin-binding cytokine that is widely expressed during early development and increases in maternal circulation during pregnancy.Aged PTN-deficient mice exhibit insulin resistance, suggesting a role in metabolic control. The objectives of this study were to determine if PTN is expressed in mouse pancreatic β-cells in young vs. adult animals, and its effects on DNA synthesis, β-cell gene expression and glucose-stimulated insulin secretion (GSIS). The *Ptn* gene was expressed in isolated fractions of young mouse β-cells, especially within immature β-cells with low glucose transporter 2 expression. Expression was retained in the adult pancreas but did not significantly change during pregnancy. PTN and its receptor, phosphotyrosine phosphatase-β/ζ, were also expressed in the proliferative INS1E β-cell line. Fluorescence immunohistochemistry showed that PTN peptide was present in islets of Langerhans in adult mice, associated predominantly with β-cells. The percentage of β-cells staining for PTN did not alter during mouse pregnancy, but intense staining was seen during β-cell regeneration in young mice following depletion of β-cells with streptozotocin. Incubation of INS1E cells with PTN resulted in an increased DNA synthesis as measured by Ki67 localization and increased expression of *Pdx1* and insulin. However, both DNA synthesis and GSIS were not altered by PTN in isolated adult mouse islets. The findings show that *Ptn* is expressed in mouse β-cells in young and adult life and could potentially contribute to adaptive increases in β-cell mass during early life or pregnancy.

## Introduction

Pleiotrophin (PTN) is a heparin-binding cytokine that is highly conserved across mammalian species (1, 2). It is expressed in multiple tissues during embryonic and fetal development and is important for tissue growth and morphogenesis in organs that undergo branching morphogenesis (3, 4). The pancreas is one such organ where branching ductal morphogenesis gives rise to endocrine, acinar and ductal cell lineages (5). In particular PTN contributes to cell proliferation and angiogenesis and, in the embryonic pancreas, *Ptn* is expressed in areas of vasculogenesis adjacent to the differentiating ductal epithelium. In mouse embryonic pancreatic primordia explants, inhibition of *Ptn* expression resulted in a failure of endocrine precursors to fully differentiate and express insulin or glucagon (6). By E11-13 *Ptn* expression becomes limited to the pancreatic epithelium and PTN is localized to the basement membranes, especially associated with islet budding from the ductal epithelium.

*Ptn* expression is retained following birth in multiple tissues and increased expression is found following tissue damage and inflammation (2, 7, 8). Conversely, *Ptn* ^-/-^ mice develop insulin resistance associated with a failure to accumulate fat in white adipose, increased lipolytic activity and systemic cytokine-induced inflammation (9). Moreover, late pregnant Ptn ^-/-^ mice are glucose intolerant and have an altered metabolism of both glucose and lipids, which is associated with a diabetogenic state in the pregnant mother. During pregnancy, PTN is expressed within the cytotrophoblasts of the placenta and possibly released into the maternal circulation since circulating levels more than doubled between first and third trimester of human pregnancy (10). Despite the association of PTN with metabolic control the presence and actions of PTN in the endocrine pancreas have not been described. Gene expression libraries of adult human or rodent pancreatic islets or isolated β-cells have often failed to detect substantial expression of PTN mRNA (11, 12). However, PTN was detected in islets of 3-month-old rats and was down-regulated in diabetic animals (13). Also, in humans PTN is one of only eleven circulating proteins to be significantly associated with advancing chronological age, suggesting a retention of expression in adult tissues (14).

A major cell membrane receptor for PTN is the receptor phosphotyrosine phosphatase-β/ζ (RPTP β/ζ) encoded by the protein tyrosine phosphatase receptor Type Z1 (PTPRZ1) gene (2). PTPRZ1 is strongly expressed in adult mouse and rat pancreatic islets, mouse β-cell lines, and in human β-cells (15), raising the possibility that β-cells may be a target for PTN actions. PTN undergoes proteolytic modification in the extracellular fluid resulting in proteins with differing biological activities. For instance, cleavage by plasmin or metalloproteinase 2 (MMP2) can result in multiple peptides which differentially alter angiogenesis and can alter the actions of VEGF-A on endothelial cells (16, 17). In addition to RPTP β/ζ, a number of other PTN-binding proteins are associated with pancreatic islets such as the αvβ3 integrin (18).

In this study we have examined the expression and presence of PTN in young and adult mouse pancreas, how this might alter during pregnancy and the actions of PTN on β-cell DNA synthesis and glucose-stimulated insulin release.

## Materials and Methods

### Ethics Approval

All animal procedures were approved by the Animal Care Committee of Western University in accordance to the guidelines of the Canadian Council of Animal Care (approval number 2018-027). Animals were housed in a temperature-controlled room with 12 h light:12 h dark cycle and water and food were given *ad libitum*. Tissue sections from formalin-fixed human pancreata were obtained from the Department of Pathology and Laboratory Medicine, Schulich School of Medicine and Dentistry, Western University, London, ON, Canada with approval from the Western University Research Ethics Board (#103167). Pancreata were examined from three healthy male and three female individuals who had died as young adults or aged 60-70 years.

### Animals

Pancreata were collected from non-pregnant female or male C57BL/6 mice (strain code 027) (n=6 per study group) (Charles River, Wilmington, MA, USA) aged 6-8 weeks of age immediately following euthanasia using an approved CO_2_ chamber. Female mice of similar age underwent estrous cycling and were time mated. Timed pregnancies were established as previously described (19) and animals euthanized at gestational days (GD) 9, 12 and 18. For histology, tissues were fixed with 4% paraformaldehyde and embedded in OCT compound (Optical Cutting Temperature, Tissue-Tek, ThermoFisher, Waltham, MA, USA) for cryosection preparation. At least two sections per pancreas (replicates), separated by >100µm, were used for immunohistochemical analysis.

In separate experiments using neonatal C57BL/6 mice animals were euthanized at 7 days of age and the pancreas removed for enzymatic dispersal prior to fluorescence-activated cell sorting (FACS) and DNA microarray analysis. In another experiment, neonatal male and female BalbC mice were treated with streptozotocin (STZ 35 mg/kg, Sigma-Aldrich, St. Louis, MO, USA) or an equal volume of vehicle (0.1M sodium citrate buffer, pH 4.5) by subcutaneous injection from days 1-5 of life (20). Animals were euthanized 14 days after the final administration of STZ at 19 days of age and pancreata were collected, fixed and prepared for histology. Blood glucose values were determined after a 2- to 4h fast using a glucometer (Ascencia Breeze, Bayer Inc., Toronto, ON, Canada). This STZ treatment regime results in an approximate 60% reduction in insulin-immunoreactive cells within islets 48h following the final injection of STZ (20).

### Immunohistochemical Analysis of Pancreas

Cryosections (7 μm thickness) of mouse pancreas and paraffin sections (5 μm) of human pancreas were prepared. Human tissue sections were deparaffinized and rehydrated in PBS. Antigen retrieval was performed in citrate buffer (10mM sodium citrate, 0.05% Tween20, pH 6.0) for 30 min at 100°C using a Decloaking Chamber (Biocare Medical, Concord, CA, USA). Following an 8 min incubation with Sniper block (Intermedico, Markham, ON, Canada) to block any non-specific binding, primary antibodies were applied against insulin (1:50 guinea pig polyclonal, Abcam, Cambridge, UK), glucagon (1:2000 mouse monoclonal, Sigma), somatostatin (1:100 mouse monoclonal, Santa Cruz Biotechnology Inc, Dallas, Texas, USA), Glucose transporter-2 (Glut2) (1:100 rat monoclonal, R&D Systems, Minneapolis, MN, USA) or PTN (1:200 rabbit polyclonal, ThermoFisher) overnight at 4°C. The following day immunofluorescence secondary antibodies (1:400 goat anti-guinea pig 555, goat anti-mouse 555, goat anti-mouse 647, donkey anti-rat 647, goat anti-rabbit 488, ThermoFisher) were applied for 2 h at room temperature. Nuclei were counterstained with DAPI (1:500 4,6-diamidino-2-phenylindole) dihydrochloride (ThermoFisher). The specificity of PTN immunostaining was determined by the absence of signal following omission of the primary PTN antibody, omission of the secondary antibody, or pre-absorption of the primary antibody overnight with excess recombinant PTN (10 μg, R&D Systems) before applying to the sections.

All insulin and glucagon-expressing cells were imaged at 20x magnification, with the observer being blind to tissue identity, using a Nikon Eclipse TS2R inverted microscope with NIS-Elements. Analysis was performed using ImageJ software and manual counting of stained cells to calculate the percentage of cells co-stained with combinations of insulin, glucagon, somatostatin and PTN, or insulin, Glut2 and PTN. Endocrine cells were classified as belonging to islets (>5 β-cells) or extra-islet endocrine clusters (1-5 β-cells) (21).

### Fluorescence Activated Cell Sorting and DNA Microarray

Dispersed cells isolated from whole pancreata of 7-day-old C57BL/6 mice following digestion with collagenase V at 37°C in a shaking water bath for 30 min were subjected to FACS as described previously (21). Antibodies against GPm6a (1:100, PE conjugated; Bioss Inc, Woburn, MA, USA) and Glut2 (1:100, 647 conjugated; Bioss) were incubated for 60 min to label β-cells. Seven-Aminoactinomycin D (7-AAD) (1:100; BD Biosciences, San Jose, CA, USA) was added as a viability marker. GPm6a is a cell surface marker specific for mouse β-cells (22). Cells were sorted on a Becton Dickinson FACSAria III cell sorter using FACSDiVa software (v 8.0.1) at the London Regional Flow Cytometry Facility, Western University, London, ON to separate insulin (GPm6a)-expressing cells co-expressing Glut2 (Ins^+^Glut2^HI^) from insulin-expressing cells with little Glut2 (Ins^+^Glut2^LO^) (21).

Total RNA was extracted and purified separately from the above Ins^+^Glut2^HI^ or Ins^+^Glut2^LO^ cell fractions separated by FACS from neonatal mouse pancreata using the RNeasy Plus Mini kit (QIAGEN, Hilden, Germany). Replicate samples were prepared for each from pooled pancreata derived from different mice. Each total RNA pool was derived from 18-20 pancreata. RNA samples from Ins^+^Glut2^HI^ or Ins^+^Glut2^LO^ cell fractions were hybridized to a GeneChip and processed at the London Regional Genomics Centre, Western University, London, ON. RNA quality was assessed using the Agilent 2100 Bioanalyzer (Agilent Technologies Inc., Palo Alto, CA, USA) and the RNA 6000 Nano kit (Caliper Life Sciences, Mountain View, CA, USA). For expression analysis, the Affymetrix (Santa Clara, CA, USA) GeneChip Mouse Genome 430 2.0 (MOE430 2.0) array was used, containing 45,000 probe sets representing transcripts and variants from 34,000 mouse genes. All procedures, including cRNA synthesis, labelling, and hybridization to Affymetrix MOE430 2.0 GeneChips, were performed as described in the Affymetrix Technical Analysis Manual. The GeneChips were scanned with the Affymetrix GeneChip Scanner 3000 and probe level data from the.CEL files were analysed using Partek Genomics Suite v6.5 (Partek, St. Louis, MO, USA). Probes were imported and summarized using multi-array averaging and ANOVA was used to determine fold changes.

### INS1E Cell Culture

The rat insulinoma cell line, INS1E, was a gift from Dr. Dawn Kilkenny, University of Toronto, ON, Canada. The cells were maintained in RMPI 1640 medium (Sigma) supplemented with 2 g/L sodium bicarbonate, 10 mM Hepes, 11.2 mM D-glucose, 1 mM sodium pyruvate, 2 mM L-glutamine, 50 µM beta-mercaptoethanol, 100U/ml penicillin/streptomycin and 10% heat-inactivated fetal bovine serum (FBS). Cells were cultured in a humidified incubator at 37°C and 5% CO_2_ and passaged when cultures reached 80% confluency. For all *in vitro* experiments, cells were plated at a density of 5x10^5^ cells/well in 6-well multiwell tissue culture plates (Falcon, VWR International, Mississauga, ON, Canada).

For analysis of DNA synthesis culture medium was changed to RPMI containing 3% FBS with or without PTN (0.1 or 1.0 μg/ml PTN-15, R&D Systems)) and cells were incubated for 24 or 48 h at 37°C. For immunohistochemical analysis, cells were cultured on coverslips in 6-well multiwell tissue culture plates and fixed with 4% paraformaldehyde for 30 min at room temperature. Fixed cells were permeabilized by treating with 0.3% TritonX-100/phosphate-buffered saline (PBS) for 10 min at room temperature, then incubated in Sniper Block for 8 min. Coverslips were washed with 0.2% TritonX/100 in PBS containing bovine serum albumin, followed by incubation with primary antibodies to insulin (1:2000 mouse monoclonal, Sigma) and Ki67 (1:200 rabbit polyclonal, Neomarkers, ThermoFisher) overnight at 4°C. The following day, coverslips were incubated for 1 h at room temperature with secondary antibodies (1:400 goat anti-mouse 555 and goat anti-rabbit 488, ThermoFisher). Nuclei were counterstained with DAPI (4,6-diamidino-2-phenylindole) dihydrochloride (1:500, ThermoFisher). Results shown represent four replicate culture wells with an average of 4 random field cell counts per coverslip for each replicate experiment.

For analysis of glucose-stimulated insulin secretion INS1E cells (n=4 replicates) were pre-incubated with glucose free Kreb’s buffer solution (KRB; 119mM NaCl, 4.7mM KCl, 25mM NaHCO_3_, 2.5mM CaCl_2_·2H_2_O, 1.2mM MgSO_4_·7H_2_O, 1.2mM KH_2_PO_4_, 1% BSA, 10mM Hepes) with or without recombinant mouse PTN (0.1 or 1.0 μg/ml) for 60 min at 37°C. Subsequently, cells were incubated with KRB supplemented with 2.8 mM or 28.8 mM glucose with or without PTN for 2 hours at 37°C. Supernatants were collected and cell monolayers were scraped and treated with acid-ethanol to extract insulin. Insulin in both supernatants and cell homogenates was measured using an Ultrasensitive Rat Insulin ELISA kit (Crystal Chem, Downers Grove, IL, USA). Data was collected using a BioRad iMark plate reader and analyzed with Microplate Management software.

### Isolated Pancreatic Islets

Pancreata from adult mice (8 weeks age) were digested with either collagenase V or liberase (Roche Diagnostics, Indianapolis, IL, USA). In the case of liberase mice were euthanized with CO_2_ and 5 ml of cold enzyme was injected into the pancreas *via* the common bile duct. The distended pancreata were removed, digested at 37°C for 12.5 min before islet separation. When collagenase was used the pancreas was dissected free at sacrifice and finally chopped before enzymic dispersal. Islets were separated from exocrine tissue using a Dextran density gradient of 27, 23 and 11% concentrations. Islets were collected from the 23/11% interface into a sterile p60 petri dish (Falcon) with 5 ml RPMI 1640 medium containing 10% FBS and 6.5 mM glucose.

For estimation of DNA synthesis islets were incubated for 24 h at 37°C and 5% CO_2_ before allocation to 6-well ultra-low attachment multiwell plate (approximately 20 islets per treatment) (Falcon) in RPMI medium for 48 h, with and without PTN (0.1 or 1.0 μg/ml). Following incubation islets were hand-picked and allowed to affix to glass-bottom dishes (MatTek Life Sciences, Ashland, MA, USA) pre-adsorbed with diluted Cell-Tak adhesive (BD Biosciences), fixed in 4% paraformaldehyde for 30 min at room temperature and stored at 4°C in PBS. Immunofluorescent staining for insulin and Ki67 was performed on whole islets as described above. Z-stack images were collected from islets using confocal microscopy starting from the base of the islets at the glass surface (Nikon A1R, Nikon Canada, Mississauga, ON, Canada) with an average of 26 images per stack over a depth of approximately 350 μm. Four to six randomly selected images per islet were analyzed using the cell counter on ImageJ software and the percentage of cells co-staining for Ki67 was calculated.

To measure glucose-stimulated insulin secretion pools of islets were pre-incubated in RPMI media containing 6 mM glucose for 24 h with or without PTN (1μg/ml). Eighteen islets were size-matched in batches and pre-incubated in 0.5 ml of Kreb’s buffer (140 mM NaCl, 5 mM KCl, 1.2 mM MgCl_2_, 2.6 mMCaCl_2_, 1 mM NaH_2_PO_4_, 5 mM NaHCO_3_, and 10 HEPES, pH 7.4) supplemented with 2 mg/ml bovine serum albumin (Sigma Aldrich) and 1 mM glucose for 1 h at 37°C to establish a baseline insulin secretion. Following pre-incubation, the islet batches were subsequently incubated in 0.12 ml of Kreb’s buffer supplemented with either 2 mM or 25 mM glucose, or 2 mM glucose in the presence of 200 μM tolbutamide (Sigma-Aldrich), for 2 h at 37°C. Following incubation, the supernatant was quickly removed and stored at -80°C until measurement of insulin content using an ELISA (described above). For total insulin islet content, the remaining islets were lysed in HCl : EtOH (1:15 v/v) at the end of the experiment, were sonicated, and stored at -80°C until insulin assay. Five replicate islet incubations were performed for each experimental variable.

### Quantitative PCR

INS1E cells (n = 6 replicates) were incubated for 24h at 37°C with complete RPMI medium containing 3% FBS with or without PTN (0.1, 1.0 µg/ml). RNA from each well was isolated using RNeasy Plus Mini Kit (Qiagen) and measured using a NanoDrop spectrophotometer (DeNovix, Waltham, MA, USA). RNA was reverse transcribed to cDNA using iScript Reverse Transcription Supermix (Bio-Rad, Hercules, CA, USA). Quantitative polymerase chain reaction (qPCR) experiments were performed after confirmation of parallel PCR amplification efficiencies on a QuantStudio 5 Real-Time PCR System (ThermoFisher). SybrGreen Master Mix (ThermoFisher) and specific primers (Sigma) (**Supplementary Table**) at a concentration of 2.5 mM were used to detect PCR products. cDNA (20 ng) was added per reaction. qPCR was performed on triplicate samples with an initial polymerase activation step at 95°C for 20 sec, followed by 40 cycles of denaturation (95°C for 3 sec) and annealing/extension (60°C for 30 sec). Relative gene expression was quantified using the ΔΔ cycle threshold (C_T_) method and calculated as fold change compared to the geometric mean of the housekeeping gene β-actin.

Quantitative PCR was also performed on RNA samples prepared from pregnant adult mouse pancreas at GD 9 to 12 and at GD 18, as well as age-matched non-pregnant mice. In this experiment a QuantStudio5 Real-time PCR System was utilized (Applied Biosystems, Waltham, MA, USA). TaqMan primers (assay numbers) for PTN (Mm01132688_m1) and for the ‘housekeeping’ control genes, cyclophilin A (cycloA, Mm02342429_g1) and glyceraldehyde-3-phosphate dehydrogenase (GAPDH, Mm99999915_g1) (TaqMan Gene Expression Assays, Applied Biosystems) were used. qPCR was performed using TaqMan Fast Advanced Master Mix (Applied Biosystems) on triplicate samples following manufacturer’s instructions. We utilized the average C_T_ value for each primer at each concentration of cDNA, determined by the Quantstudio Design and Analysis Software (Thermo Fisher). ΔC_T_ values were then calculated by subtracting the C_T_ of each housekeeping gene from the C_T_ of the PTN primer of interest at each cDNA concentration. These ΔC_T_ values were graphed against the log of the cDNA concentration. The absolute value of the slope had to be <0.1 in order to demonstrate that the efficiencies of target and reference genes were approximately equal.

### Statistical Analysis

Data are presented as mean ± SEM with between 4-12 replicates per experiment using Graph Pad software. A two-tailed Student’s t-test, paired two-tailed Student’s t-test, one-way ANOVA or two-way ANOVA were applied according to the experimental design. A Tukey’s *post-hoc* test or a Bonferroni *post-hoc* test was performed after one-way ANOVA or two-way ANOVA analysis, respectively. Statistical significance was determined as p<0.05.

## Results

**Figure 1:**
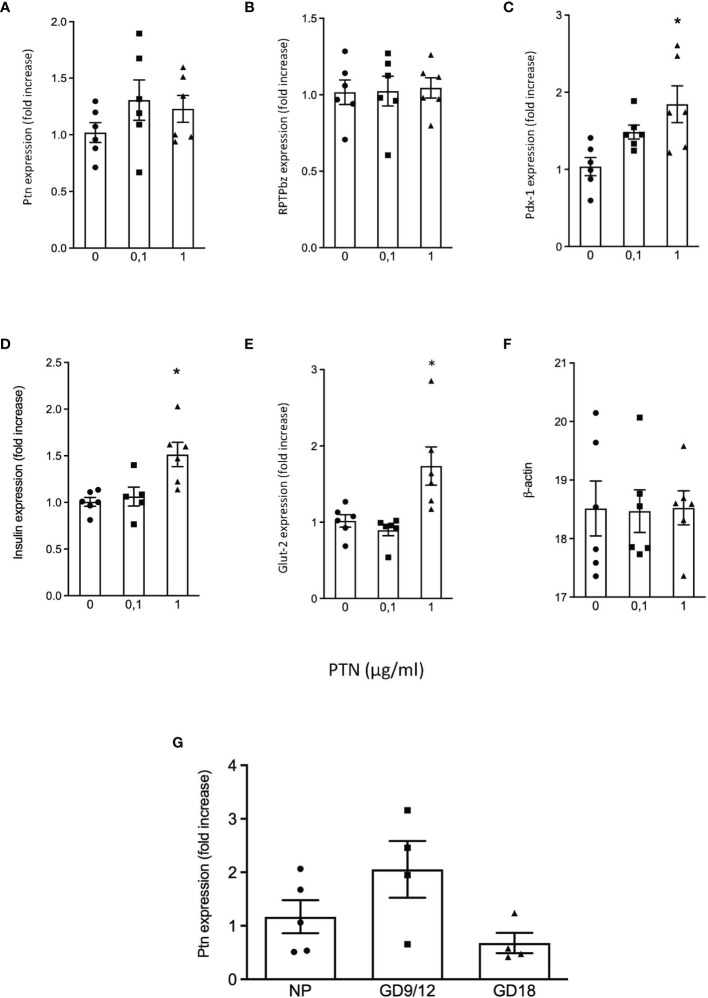
Quantification of mRNA expression in INS1E cells treated without or with 0.1 μg/ml or 1 μg/ml PTN. Fold change vs. control without PTN for **(A)** *Ptn*, **(B)** *Ptprz1* (RPTP β/ζ), **(C)** *Pdx1*, **(D)** *Ins* (insulin), **(E)** *Glut2* and **(F)** β actin which served as a ‘housekeeping’ gene. *p < 0.05 vs. control (n=6). The relative expression of *Ptn* in adult mouse pancreas in non-pregnant mice, and pregnant mice at GD 9/12 and at GD 18 is shown in **(G)**, n=4-5.

We examined β-cells isolated by FACS from the young mouse pancreas for *Ptn* and *Ptprz1* mRNA presence and found both (**Table 1**). Previously, we had identified a sub-population of β-cells in mouse and human pancreas that have little Glut2 expression but have greater proliferative potential (21). Thus, we examined if a differential expression of *Ptn* and *Ptprz1* existed between Ins^+^Glut2^LO^ and the more abundant and functional Ins^+^Glut2^HI^ cells. *Ptn* was five-fold more abundantly expressed within Ins^+^Glut2^LO^ cells and *Ptprz1* expression three-fold greater when compared to Ins^+^Glut2^HI^ cells (**Table 1**). Other PTN binding proteins differentially expressed between Ins^+^Glut2^LO^ and Ins^+^Glut2^HI^ cells were the αvβ3 integrin sub-units, and heparin sulphate proteoglycan 2 (*Hsgp2*). Messenger RNA for PTN and RPTP β/ζ was also readily detectable in the INS1E β-cell line (**Figure 1A, B**), when compared to the house-keeping gene β-actin (**Figures 1F**), and *Ptn* continued to be expressed in adult mouse pancreas (**Figure 1G**).

**Table 1:** Fold increase in selected genes related to PTN presence or action in Ins^+^Glut2^LO^ cells relative to Ins^+^Glut2^HI^ cells isolated from pancreata of young mice (postnatal day 7).

Gene Name	Protein Name	Fold Increase
*Ptn*	Pleiotrophin	4.9
*Ptprz1*	Protein tyrosine phosphatase receptor Type Z1	2.9
*Adamts1*	ADAM Metallopeptidase with Thrombospondin Type 1 Motif 1	12.6
*αv integrin*	Alpha v integrin	5.5
*β3 integrin*	Beta 3 integrin	3.6
*Hspg2*	Heparan sulfate proteoglycan 2	25.9

**Figure 2:**
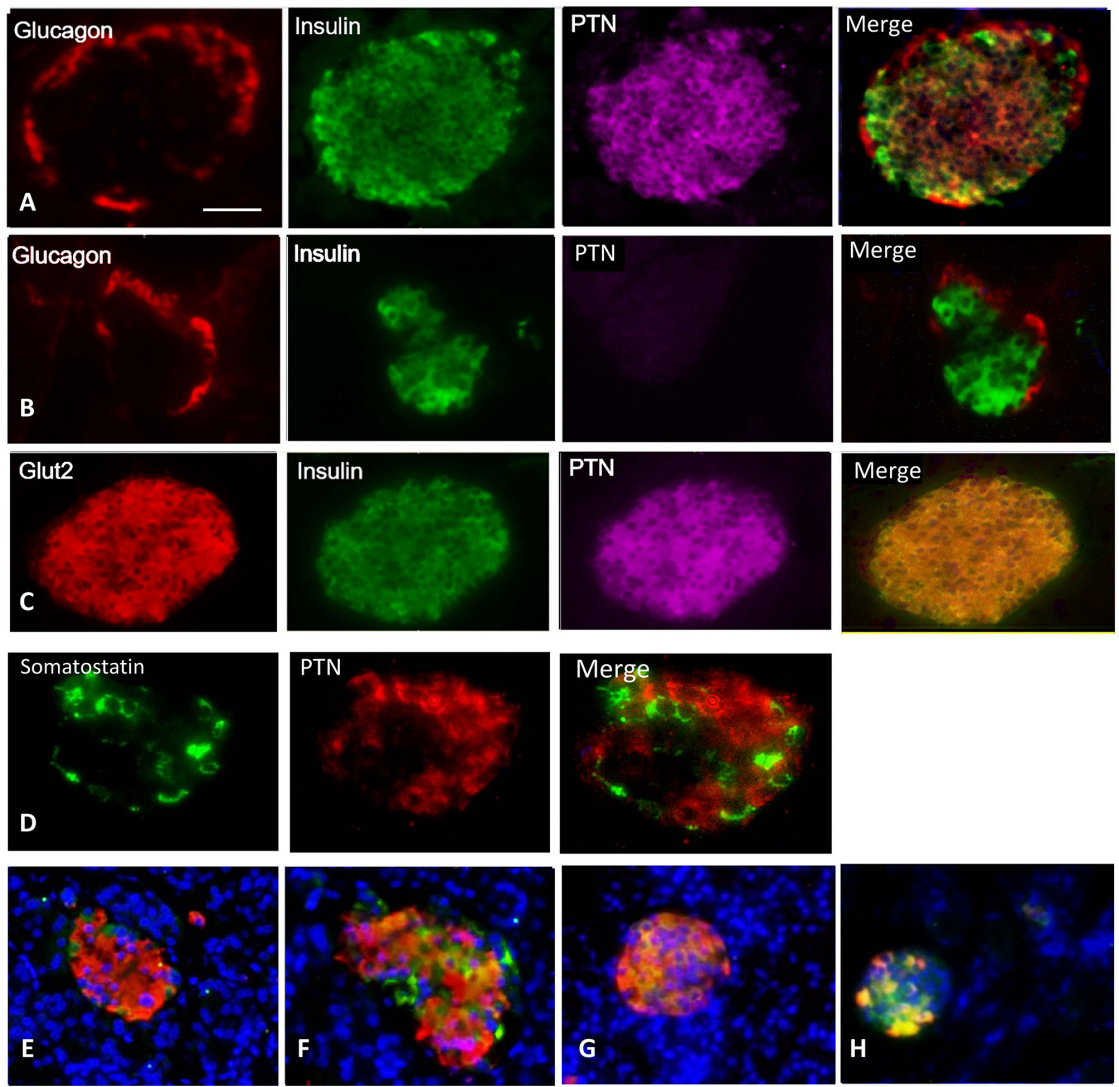
Representative images for: **(A)** Immunohistochemical localization of glucagon (red), insulin (green), PTN (magenta), and merged image of an adult male mouse islet. **(B)** Control staining is shown for a separate islet where the primary antibody for PTN was omitted. **(C)** A female mouse islet showing co-localization of Glut2 (red) with insulin (green) and the presence of PTN (magenta) in β-cells. **(D)** A female mouse islet showing the distribution of somatostatin (green) and PTN (red). **(E, F)** Two different sections of a human pancreas from a male age 67 years showing insulin (red), PTN (green) and nuclei counterstained with DAPI (blue). **(G)** Control mouse pancreas, and **(H)** mouse pancreas 14 days following administration of streptozotocin to partially destroy β-cells, showing insulin (red) and PTN (green). Bar represents 100 μm in A-C, 50 μm in D, and 80 μm in **(E–H)**.

Since RPTP β/ζ was expressed by β-cells, PTN may be part of the cell surface proteome in adult mouse through sequestration by the receptor or other binding proteins in addition to a local expression. PTN was localized within adult mouse pancreas sections by fluorescence immunohistochemistry. PTN immunoreactivity was absent from the acinar tissue and pancreatic ducts but was present in the islets of Langerhans and smaller extra-islet endocrine clusters. Staining was predominantly localized to β-cells within islets, as judged by cellular co-localization with insulin (84.7 ± 2.5% of β-cells, n=6 mice), and was less abundant in α-cells assessed from the co-localization with glucagon (**Figure 2A**). The specificity of the PTN staining was confirmed by the absence of signal following omission of the primary PTN antibody (**Figure 2B**), omission of the secondary PTN antibody, or prior pre-absorption of the primary antibody with excess recombinant PTN. Substantial co-localization was observed between Glut2 and PTN within islets suggesting a presence in mature β-cells (**Figure 2C**) while little co-localization was seen between somatostatin, as a marker of δ-cells, and PTN (**Figure 2D**). PTN was also observed to co-localize with β-cells within the islets of human pancreas (**Figures 2E, F**), with immunopositive cells being uniformly distributed throughout the islets. However, the presence of PTN-immunopositive cells was lower in human islets than in mouse and differed depending on whether the islets were small or larger (small < 10,000 µm^2^, 60.1 ± 2.3% of islet cells; large >10,000 µm^2^, 34.9 ± 3.2%, p<0.001). These results suggest that PTN is present on pancreatic β-cells in both adult mouse and human.

**Figure 3:**
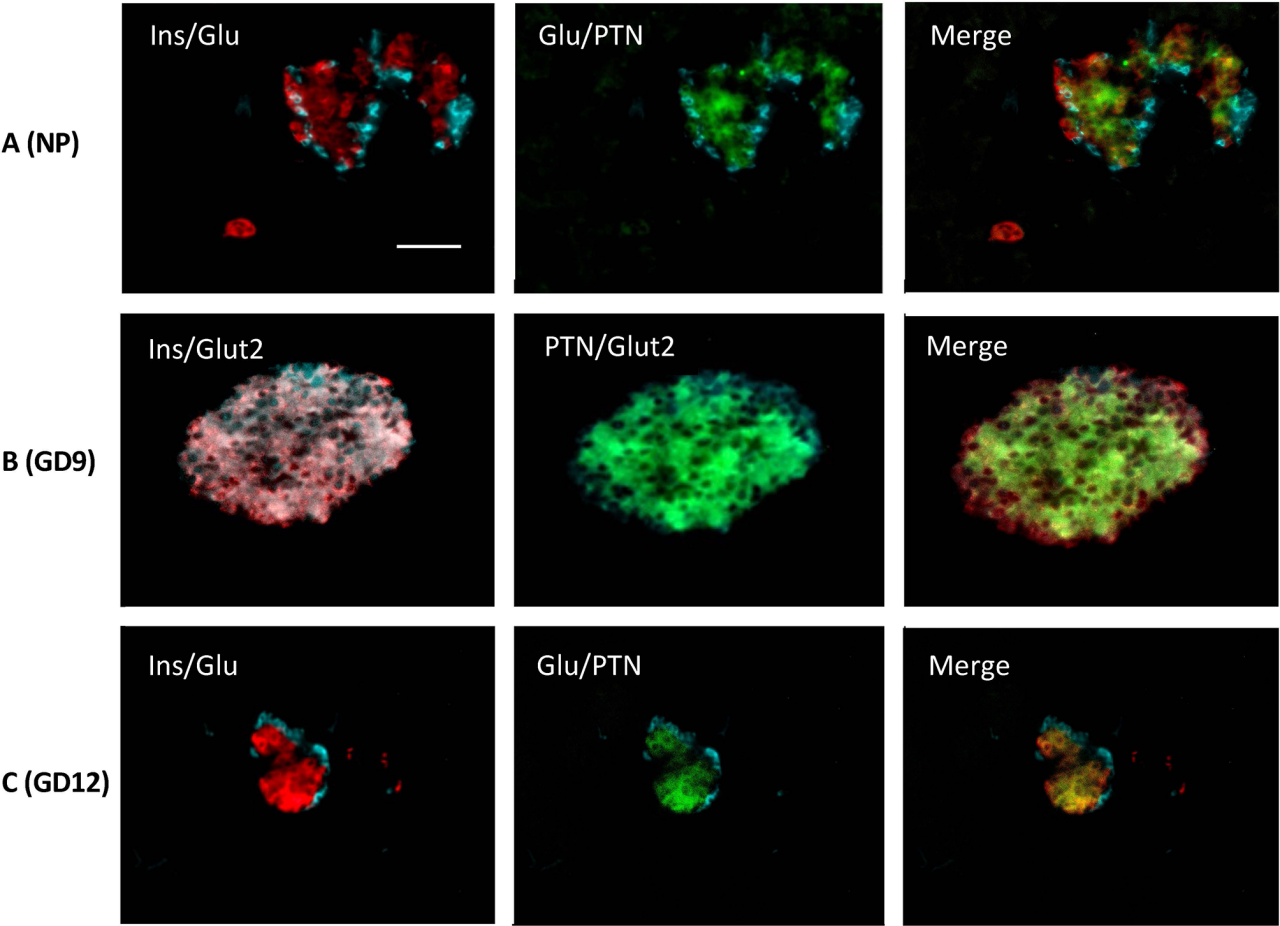
Representative images showing immunohistochemical staining in adult mouse pancreas from non-pregnant animals (**A**, NP), and during pregnancy at GD 9 **(B)** and GD 12 **(C)**. In **(A, C)** co-localization is shown left for insulin (red) and glucagon (cyan), and middle for glucagon (cyan) and PTN (green). In **(B)** co-localization is shown left for insulin (red) and Glut2 (cyan), and centre for PTN (green) and Glut2 (cyan). Merged images are shown right. Bar represents 100 μm.

We next examined if PTN presence was altered in pancreas during the mild metabolic stress represented by pregnancy. Quantitative PCR showed that the expression of *Ptn* did not significantly change over the course of pregnancy (**Figure 1G**). Pregnant mouse pancreata at GD 9, 12 and 18 were also co-stained for insulin, glucagon, Glut2 and PTN compared with non-pregnant animals (**Figure 3**). A similar co-localization of PTN to β-cells was observed during pregnancy with the percentage of β-cells that co-stained for PTN not differing from the 85% calculated in non-pregnant animals at GD 9 or 12 (n=6). At GD 9 and 12 (**Figures 3B, C**), when total β-cell mass is beginning to increase to counter the insulin resistance of pregnancy (23), PTN was largely localized to the mature β-cells expressing insulin and Glut2 (**Figure 3B**). However, PTN was also localized to immature insulin-immunoreactive cells that lacked staining for Glut2 (Ins^+^Glut2^LO^ cells) (**Supplementary Figure 1**).

**Figure 4:**
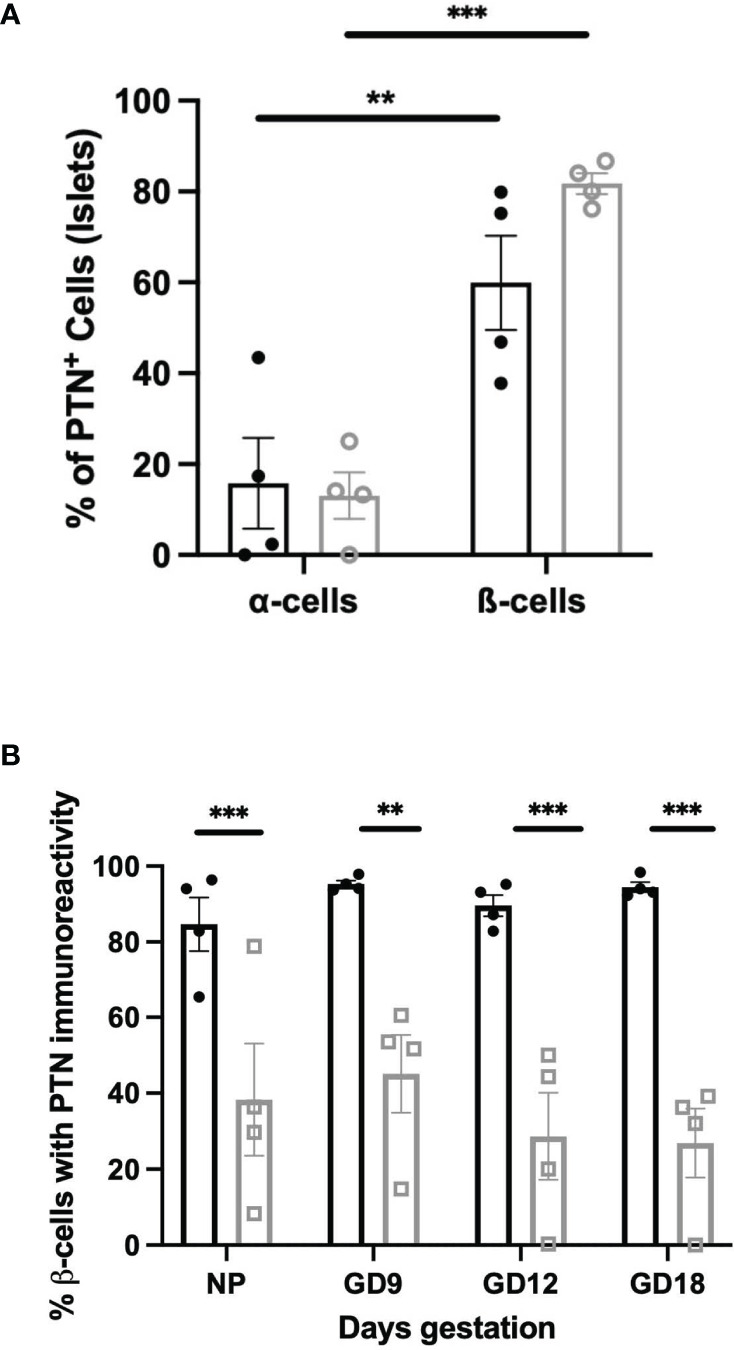
**(A)** Co-localization of PTN with α- or β-cells based on glucagon or insulin immunoreactivity respectively within sections of the female mouse pancreas. Results are shown as % of total α- or β-cells per islet that are PTN-positive in non-pregnant (black) or GD 12 (grey) mouse pancreas. **p < 0.01, ***p < 0.001 vs. α-cells. **(B)** Proportional co-localization of PTN in either Ins^+^Glut2^HI^ (black) or Ins^+^Glut2^LO^ (grey) cells within non-pregnant female mice (NP) or pregnant mice at gestational days (GD) 9, 12 or 18. **p < 0.01, ***p < 0.001 vs. Ins^+^Glut2^HI^ cells. (n=4).

The relative abundance of PTN-containing β-cells compared to α-cells was quantified in the pancreas of non-pregnant vs. pregnant female mice. Approximately 60% of PTN-immunoreactive cells within islets co-stained with insulin and 20% with glucagon in non-pregnant animals (**Figure 4A**). This did not differ at GD 12 of pregnancy. These results show that the distribution of immunoreactive PTN between pancreatic endocrine cell types did not significantly differ during the adaptive increase in β-cell mass that occurs during mouse pregnancy. Further, we examined the proportion of β-cells within islets that contained PTN immunoreactivity when Ins^+^Glut2^HI^ and Ins^+^Glut2^LO^ cells were considered separately, and how this might change across pregnancy. This relative distribution of PTN between β-cell phenotypes did not significantly change over the course of pregnancy (**Figure 4B**).

To examine a more pronounced metabolic stress than pregnancy, we partially depleted β-cells by treatment with STZ. Two weeks following STZ administration the number of insulin-staining β-cells were substantially reduced compared to control mice, a time point associated with a regenerative response of β-cells in young animals (20). These animals still had relatively elevated mean blood glucose values at this time relative to vehicle-treated controls (STZ 11.9 ± 1.4 mM, control 8.4 ± 0.6 mM, p<0.05). Strong PTN immunoreactivity was associated with the remaining β-cells compared with islets from vehicle-treated mice (**Figures 2G, H**). However, the proportion of β-cells that contained immunoreactive PTN did not differ after STZ treatment (control 88.7 ± 2.5%, STZ 90.5 ± 1.6%, n=8, 3 pancreatic sections per animal).

**Figure 5:**
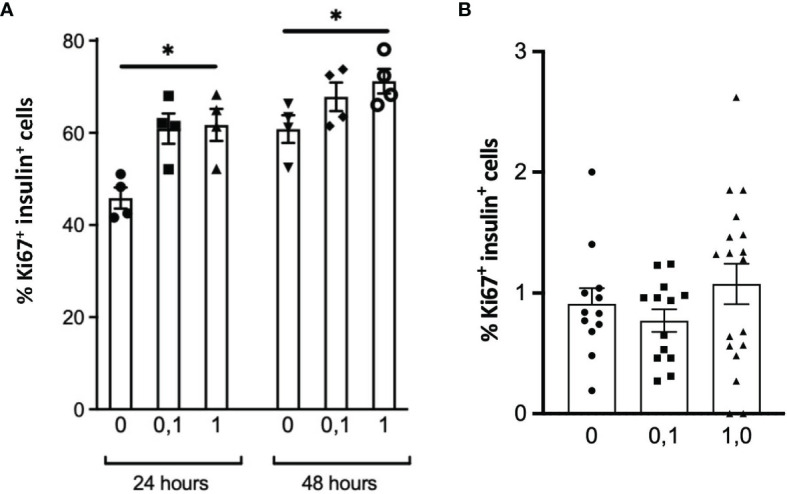
DNA synthesis in INS1E cells **(A)** or β-cells within isolated adult mouse islets **(B)** treated without or with 0.1 or 1 μg/ml PTN for 24 h or 48 h **(A)**, or 48 h **(B)** as measured from the co-localization of insulin and Ki67 by immunohistochemistry. *p < 0.05 vs. control without PTN. [n=4 replicates/experiment in **(A)**, n=11-18 separate islets per treatment in **B**].

**Figure 6:**
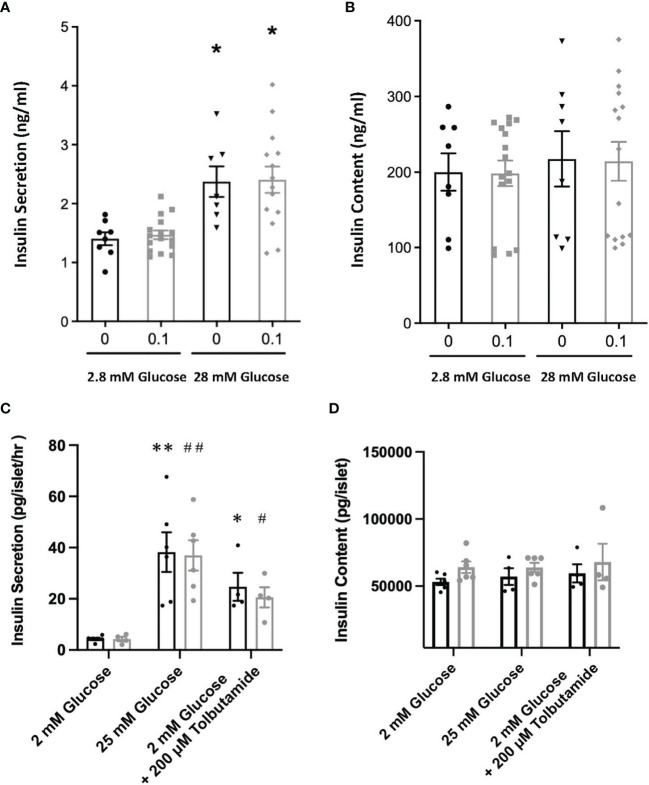
Glucose stimulated insulin release **(A)** and cellular content of insulin **(B)** from INS1E cells pre-treated with or without 0.1 μg/ml PTN following incubation with either 2.8 mM or 28.8 mM glucose. *p < 0.05 vs. 2.8 mM glucose (n=8-12). Glucose stimulated insulin release **(C)** and cellular content **(D)** of insulin from isolated mouse islets of Langerhans pre-incubated with (grey) or without (black) 1 μg/ml PTN and following incubation with either 2 mM glucose, 2 mM glucose with tolbutamide, or 25 mM glucose. *p < 0.05, **p < 0.01 vs. 2 mM glucose without PTN, and (n=6). and ^#^p < 0.05, ^##^p < 0.01 vs. 2 mM glucose with PTN (n=6).

The possibility that PTN might contribute to β-cell proliferation was examined using the INS1E cell line, a model of active β-cell replication as occurs *in vivo* in early life (20). Incubation with PTN (0.1 and 1μg/ml) resulted in a significant increase in the number of cells undergoing DNA synthesis as determined by immunoreactivity for Ki67 (**Figure 5A**). Analysis of mRNA expression using qPCR in INS1E cells showed a significant increase in the expression of the transcription factor *Pdx1* and of insulin and *Glut-2* mRNA following incubation with PTN (**Figures 1C–E**). The expression of Pdx1 was chosen since it is mechanistically associated both with β-cell proliferation as well as insulin biosynthesis. Although both *Ptn* and *Ptprz1* were expressed by INS1E cells their expression was not altered in the presence of exogenous PTN (**Figures 1A, B**). The increased expression of insulin suggested a possible additional action on insulin release. This was explored by measuring basal and glucose-stimulated insulin release from INS1E cells following incubation with PTN, and the remaining cellular content of insulin. Incubation in the presence of 28.8 mM glucose increased insulin release, but without changing the remaining cellular content, compared to 2.8 mM glucose. However, neither insulin release nor content were altered in the presence of 0.1 μg/ml PTN (**Figure 6**). Thus, it appears that exogenous PTN did not alter glucose-stimulated insulin secretion from INS1E cells.

We further investigated whether PTN could alter either DNA synthesis or glucose-stimulated insulin release from isolated adult mouse islets. However, PTN (0.1 and 1μg/ml) had no significant effect on the percentage of β-cells undergoing DNA synthesis (**Figure 5B** and **Supplementary Figure 2**). Islets were also pre-incubated with 1 μg/ml PTN before exposure to either a low (2 mM) or high (25 mM) glucose stimulus *in vitro*. A seven-fold increase in insulin release was observed with 25 mM glucose compared to 2 mM, but neither the basal nor glucose-stimulated insulin release was altered by pre-incubation with PTN (**Figure 6C**). When islets were incubated with 2 mM glucose in the presence of the sulfonylurea tolbutamide, as a positive control for insulin release, a five-fold increase was observed with or without PTN. The insulin content of islets following glucose challenge was no different in the presence of PTN or tolbutamide, indicating a substantial reserve of cellular insulin that was not altered in the presence of PTN (**Figure 6D**). The findings support a lack of effect of PTN on acute glucose-stimulated insulin release. Also, in the adult mouse pancreas where the rate of β-cell turnover is low (0.2%, **Figure 5B**) compared to INS1E cells, PTN alone was unable to provide a mitogenic stimulus.

## Discussion

The presence of PTN was localized to the islets of Langerhans of adult male and female mice and also in human pancreas. All sizes of islets showed a predominant co-localization of PTN with insulin, although a minority showed co-localization to glucagon-containing α-cells. Little staining was associated with δ-cells. The strong association between PTN and β-cells was further supported by the co-distribution with cells expressing *Glut2*. Although *Ptn* has not been shown previously to be prominently expressed in adult β-cells (11, 12), unlike the readily detectable levels of mRNA in early life and in proliferative β-cell lines, we show here that PTN mRNA is detectable in the adult mouse pancreas. The presence of PTN as part of the adult β-cell surface proteome could also be related to the abundant expression of the *Ptprz1* receptor (15), as well as an association with other cell surface binding proteins such as HSPG2 and the αvβ3 integrin. The distribution of PTN-immunoreactive β-cells throughout human islets corresponds to the more uniform distribution of β-cells as compared with rodents (24). However, significant differences were found in the proportion of human islet cells containing PTN depending on islet size, with PTN-positive cells being more abundant in smaller islets. This mirrors a similarly greater proportional presence of β-cells in smaller vs. larger islets in humans (25), although the percentage of islet cells represented by β-cells overall is lower than in mouse islets.

We and others have previously described a sub-population of β-cells that in mouse and human express insulin but little Glut2 glucose transporter, and therefore exhibit poor glucose-stimulated insulin secretion (21, 26, 27). These Ins^+^Glut2^LO^ cells have multi-potential islet endocrine cell lineage potentiality, and a continued proliferative capacity. Furthermore, they are more abundant during the β-cell mass expansion that occurs during pregnancy (23) and during the limited regeneration of β-cells that occurs following their depletion with STZ in young mice (21, 23). *Ptn* was expressed at five-fold higher levels in Ins^+^Glut2^LO^ cells than in the presumably more functionally mature Ins^+^Glut2^HI^ cells in the young mouse, and a similarly elevated expression of the *Adamts1* protease was seen, which cleaves PTN into a variety of bioactive fragments (28), and an increased expression of the *Ptprz1* receptor, *Hspg2* and subunits of the αvβ3 integrin. The visualization by immunohistochemistry of PTN in Ins^+^Glut2^LO^ cells as well as mature β-cells suggests that Ins^+^Glut2^LO^ progenitor cells may represent a focal point of PTN expression and paracrine action within pancreas in early life.

When mice were exposed to the metabolic stress of pregnancy there were no significant changes in the relative pancreatic expression, or the distribution of PTN between sub-populations of β-cells, despite the 2-3-fold increase in β-cell mass that occurs over the time course of normal mouse pregnancy (23). However, since the circulating levels of maternal PTN increase with the progression of pregnancy, at least in human (10), the PTN available for receptor activation on β-cells during pregnancy may include peptide derived from other tissues. The presence of PTN has been linked to tissue damage and subsequent repair, and its expression is upregulated in macrophages by inflammatory cytokines such as IFN-β and -γ (29, 30). Following depletion of β-cells with STZ in young mice intense staining for PTN was associated in the remaining β-cells 14 days following administration of STZ, at a time when β-cell regeneration is occurring (21).

What therefore might the biological actions of PTN on β-cells comprise? The turnover of β-cells in the adult mouse outside of pregnancy is slow and regeneration following β-cell loss is limited. We did not find an effect of PTN alone on the DNA synthetic rate in adult mouse β-cells. However, the increase in β-cell mass during pregnancy requires prior activation of the prolactin receptor by lactogenic hormones which cannot easily be reproduced in short-term cultures of isolated islets. It is therefore possible that in β-cells already ‘primed’ for replication PTN could have a contributory action. This is supported by the increased DNA synthesis seen when INS1E mouse β-cells, that have an endogenous replicative capacity, were incubated with PTN, and this was associated with an increased expression of insulin and *Pdx1*, the latter being required for β-cell proliferation. The increased expression of insulin and Glut-2 suggested that PTN might also modulate insulin release but no change in glucose-stimulated insulin release was found in the presence of PTN. Similarly, incubation in the presence of PTN did not alter glucose-stimulated insulin release from isolated mouse islets *in vitro*, or the islet content of insulin.

A previous study using *Ptn* knock-out mice showed these animals to become insulin-resistant in later life with an associated increase in plasma insulin levels at 12 months age (9), but that glucose metabolism was comparable to wild type controls at earlier ages. This suggests that PTN presence is not essential for insulin biosynthesis and release in adult life. Moreover, late pregnant (GD18) *Ptn* knock-out mice are hypoinsulinemic, hyperglycemic and glucose intolerant and have an altered liver metabolism of both glucose and lipids, which is associated with a diabetogenic state in the pregnant mother (31). The present study using animals less than three months of age further shows that the addition of exogenous PTN was unable to increase insulin release from isolated islets of a β-cell line. The increased levels of insulin mRNA in INS1E cells following exposure to a high dose of PTN may not represent a physiological response since circulating levels are two orders of magnitude lower, at least in the human circulation (10). The local concentration of PTN within the islets as a result of endogenous expression is not known. These findings suggest that the actions of PTN in islets are more likely to be supportive of β-cell replication, as seen when β-cell mass is expanding prior to adulthood, or during pregnancy, rather than insulin release. This agrees with actions in other tissues such as an ability of PTN to increase the number of hematopoietic stem cells (32), microglia (33) and the growth of hepatic and multiple other cancers (7).

While the intracellular pathways enabling PTN-induced β-cell replication are not known a number of pathways have been demonstrated for other cell types. The full length PTN-18 peptide was shown to enhance cell migration whilst a shorter form of PTN, PTN-15 activated mitogenesis in glioblastoma cells (34). PTN-induced cell replication in endothelial cells has been shown to involve both mitogen-activated protein kinase (MAPK) and the phosphatidylinositol 3-kinase (PI3K)-Akt pathways (35). Also, in glioblastoma cells PTN has been shown to cause a dephosphorylation of PTPRZ1, which is normally constituently active, resulting in an increase in phosphorylated β-catenin. This resulted in increased levels of cytoplasmic β-catenin, translocation to the nucleus, and binding to the transcription factor TCF/LEF with a subsequent expression of Myc and cyclin D1-enabled entry into the cell replication cycle (36).

In summary, the present findings show that *Ptn* is expressed within the endocrine pancreas in the young mouse, predominantly within β-cells, and pancreatic expression is retained in adults. PTN protein is present on β-cells, possibly by interaction with the RPTP β/ζ receptor or other PTN-binding molecules and has the potential to contribute to β-cell expansion.

## Data Availability Statement

The original contributions presented in the study are included in the article/**Supplementary Material**. Further inquiries can be directed to the corresponding author.

## Ethics Statement

The studies involving human participants were reviewed and approved by the Western University. The patients/participants provided their written informed consent to participate in this study.

## Author Contributions

The studies were conceived and planned by JS, TH, MR-A and DH. Experimental work was undertaken by AL, BS, SS and TH. All authors contributed to the writing and review of the manuscript. The present address of TH is the Oxford Centre for Diabetes, Endocrinology and Metabolism, Radcliffe Department of Medicine, Churchill Hospital, Oxford OX3 7LE, United Kingdom. All authors contributed to the article and approved the submitted version.

## Funding

This work was supported by grants from the Lawson Foundation, Toronto, ON, Canada, by the Canadian Institutes of Health Research (MOP-15263), by the Ministerio de Economía y Competitividad of Spain (RTI2018-095615-B-I00) and by Comunidad de Madrid (B2017/BMD- 3684). SS was a recipient of the Ontario Graduate Scholarship. JS was a recipient of CEU-Santander mobility grant.

## Conflict of Interest

The authors declare that they have no conflicts of interest that could be perceived as prejudicing the impartiality of the research reported.

## Publisher’s Note

All claims expressed in this article are solely those of the authors and do not necessarily represent those of their affiliated organizations, or those of the publisher, the editors and the reviewers. Any product that may be evaluated in this article, or claim that may be made by its manufacturer, is not guaranteed or endorsed by the publisher.
