# Supplementary figures and images for: Pleiotrophin Expression and Actions in Pancreatic β-Cells
Source: Front Endocrinol (Lausanne). 2022 Feb 18;13:777868. doi: 10.3389/fendo.2022.777868 (PMC8894601; doi:10.3389/fendo.2022.777868)

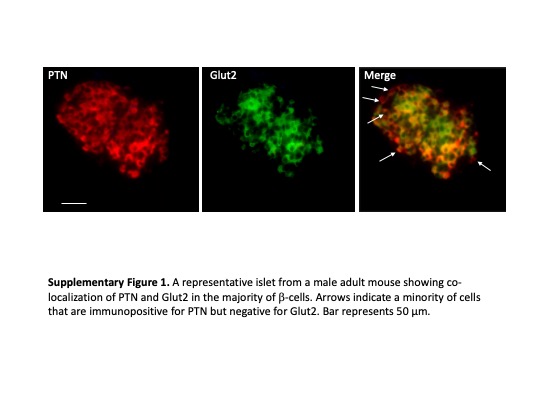

Supplement: Supplementary file 1 [file Image_1.jpeg]

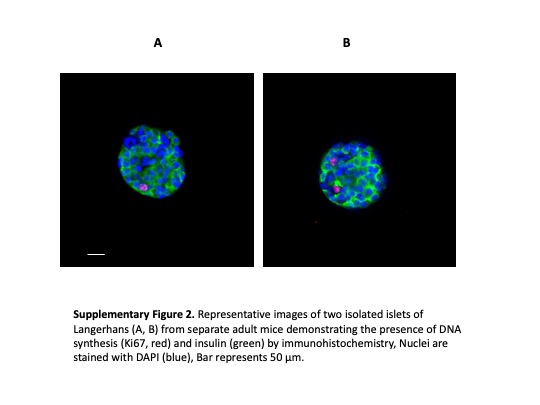

Supplement: Supplementary file 2 [file Image_2.jpeg]
