# Supplementary material for: Pleiotrophin Expression and Actions in Pancreatic β-Cells
Source: Front Endocrinol (Lausanne). 2022 Feb 18;13:777868. doi: 10.3389/fendo.2022.777868 (PMC8894601; doi:10.3389/fendo.2022.777868)
Supplement: Supplementary file 3 [file Table_1.docx]

**Supplementary Table 1. Primer sequences used for quantitative PCR analyses**

Insulin forward TCACACCTGGTGGAAGCTC

Insulin reverse ACAATGCCACGCTTCTGC

Glut2 forward CAAGATCACCGGAACCTTGC

Glut 2 reverse ATTCCGCCTACTGCAAAGCT

βactin forward GCCCCTCTGAACCCTAAG

βactin reverse CATCACAATGCCAGTGGTA

Pdx1 forward CCACACAGCTCTACAAGGACC

Pdx1 reverse CGTTGTCCCGCTACTACGTTTC

PTN forward TTGGGGAGAATGTGACCTCAATAC

PTN reverse GGCTTGGAGATGGTGACAGTTTTC

RPTPBZ forward TTTTCCAGGCAGGTGTTTTC

RPTPBZ reverse TCAGCTTGCACATTTTCTGG
